# Multiscale characterization of a lithium/sulfur battery by coupling *operando* X-ray tomography and spatially-resolved diffraction

**DOI:** 10.1038/s41598-017-03004-4

**Published:** 2017-06-05

**Authors:** Guillaume Tonin, Gavin Vaughan, Renaud Bouchet, Fannie Alloin, Marco Di Michiel, Laura Boutafa, Jean-François Colin, Céline Barchasz

**Affiliations:** 1 Univ. Grenoble Alpes, CEA, LITEN, DEHT, LM, Grenoble, F-38000 France; 2 European Synchrotron Radiation Facility (ESRF), Grenoble, 38000 France; 3 Univ. Grenoble Alpes, LEPMI, F-38000 Grenoble, France CNRS, LEPMI, 38000 Grenoble, France; 4 Réseau sur le Stockage Electrochimique de l’Energie (RS2E), CNRS, FR3459, 33 Rue Saint Leu, 80 039 Amiens Cedex, France

## Abstract

Due to its high theoretical specific capacity, the lithium/sulfur battery is one of the most promising candidates for replacing current lithium-ion batteries. In this work, we investigate both chemical and morphological changes in the electrodes during cycling, by coupling *operando* spatially resolved X-ray diffraction and absorption tomography to characterize Li/S cells under real working conditions. By combining these tools, the state of the active material in the entire cell was correlated with its electrochemical behavior, leading to a deeper understanding of the performance limiting degradation phenomena in Li/S batteries. Highly heterogeneous behavior of lithium stripping/plating was observed in the anode, while the evolution of sulfur distribution in the cathode depth was followed during cycling.

## Introduction

The increasing market for electric vehicles requires development of new energy storage systems. One of the most promising technologies is the lithium/sulfur (Li/S) battery^1–3^, which presents high theoretical energy density (2500 Wh.kg^−1^)^4^, and is based on abundant and low cost active material. However, the complex mechanisms^5, 6^ which the battery undergoes during cycling are still unclear and depend on the system (electrode morphology, loading, solvent and additives used in the electrolyte). Contrary to the insertion/disinsertion^7, 8^ mechanism of conventional lithium-ion batteries (LIBs), Li/S cells transfer charge *via* a series of complex chemical and electrochemical coupled reactions^9^ involving dissolution and precipitation of sulfur species. Capacity fading in these systems typically occurs after only a few cycles with stabilization of the capacity far below the theoretical value due to several phenomena, in particular to the positive electrode failure. Some groups have reported capacity fading due to morphological changes^10^ in the electrode, the electrically insulating nature of sulfur and Li_2_S^5^ and the solubility of active species inducing a shuttle mechanism^11^. In addition, the use of a lithium metal electrode also limits the cyclability^12^, as plated and stripped lithium has weak coulombic efficiency and poor morphological reversibility during cycling, with volume variation due to the inhomogeneous plating^13, 14^ and solid electrolyte interphase (SEI) formation^15, 16^. Understanding the mechanisms within the cell and the behavior of both electrodes is thus crucial for improving the performance of cell components and thus the economic viability of the system. The aim of this work is to perform *operando* characterization of batteries in order to better understand the failure mechanisms of Li/S cells and the important parameters governing these processes.

X-ray diffraction (XRD) has been used by many groups to characterize the formation and consumption of crystalline active species^17–20^ in these systems. However, most of studies were performed *ex*-*situ* ^17^. Recently, *in situ* ^18^ and *operando* XRD has been shown to be an excellent probe to study microstructural changes in Li/S cells^19, 21, 22^, although these studies lacked spatial resolution. Other techniques^11^, such as X-ray absorption spectroscopy^9, 23, 24^, or UV/Vis spectroscopy^25^ allow in principle the characterization of polysulfides composition *in situ*, whereas X-ray absorption tomography allows changes in the global morphology of the electrodes to be studied upon cycling. In the last decade, several groups have applied X-ray absorption tomography^26–28^ to Li-ion batteries, although often without *operando* characterization. In Li/S system, Zielke *et al*.^10^ were able to probe sulfur penetration into the carbon-based electrode *ex situ* using X-ray absorption tomography. More recently, Risse *et al*.^29^ performed *operando* radiography of Li/S cells and discussed evolution of the macroscopic structure.

In the broader field of materials science, experiments combining X-ray tomography and X-ray diffraction have already been carried out in order to characterize materials at multiple length scales^30–32^. Pietsch *et al*.^33^ combined X-ray tomographic microscopy and scanning X-ray diffraction in order to follow dynamic processes, in particular the dynamic distribution of the previously reported Li_15_Si_4_ phase in silicon-based lithium-ion batteries. However, to our knowledge, here we present for the first time *operando* X-ray tomography coupled with *operando* and spatially resolved XRD diffraction study applied to the Li/S cell. The aim is to obtain semi-quantitative information on the chemical and morphological states of the battery while cycling, from the microscopic to the macroscopic length scale and for the different compounds within the Li/S cell. The consumption and deposition processes of metallic lithium, lithium sulfide and sulfur have been characterized during two charge/discharge cycles.

## Results

**Figure 1:**
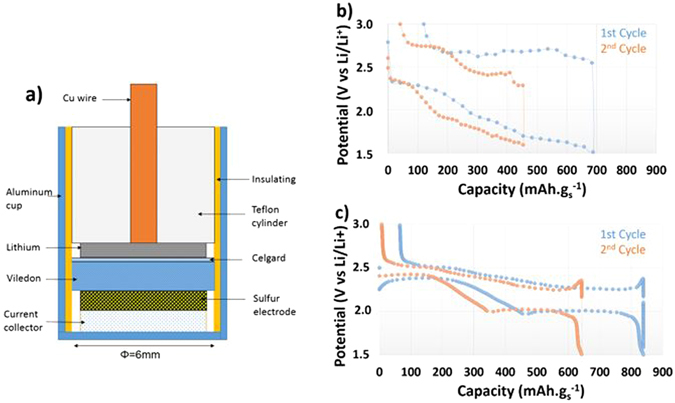
(**a**) Schematic of *operando* cell. (**b**) Voltage profile of *operando* Li/S cell. Each points corresponds to a tomography measurement. (**c**) Typical voltage profile in a CR2032 Li/S coin cell.

The cell design used for the electrochemical cycling and *operando* characterizations of the Li/S battery is shown schematically in Fig. 1a. The design has been adapted from a typical coin cell, in order to have the necessary small cross-section required for high-resolution tomography measurements, and rotational symmetry useful for the XRD characterization. A typical voltage profile obtained with this cell at constant current is shown in Fig. 1b, each values correspond to a tomographic point. The profiles are compare favorably to the expected profiles in coin cells, as shown in Fig. 1c. Although larger polarization and some noise are observed with the *operando* cell due to poor electrical contact while rotating the cell during tomography measurements, we observe the expected two plateaus with capacity values in the same range of magnitude as obtained in coin cells. During recharging, a quasi-reversible process occurs.

The battery was housed in an aluminum container, selected for its mechanical properties, the relatively thin wall thicknesses, and the possibility to use the casing as a current collector of the positive electrode. Porous non-woven carbon paper (NwC) was preferred to aluminum foil for supporting the positive electrode, due to better performance^10^ in terms of charge/discharge capacity, capacity retention and higher accessible sulfur loading. The cell design for the experiment was chosen to be as close as possible to a real Li/S cell, while being small enough to make X-ray diffraction and tomography feasible at reasonable incident beam energies.

The cell was mounted on beamline ID15A at the ESRF, and was sequentially studied by absorption tomography and spatially resolved diffraction with a recording frequency for one full measurement (diffraction plus tomography) of approximately 20 min. During the measurements the cell was cycled. The first cycle was performed at the theoretical rate of C/20 (8 hours in practice, due to incomplete utilization of the active material) and the second at C/40.

**Figure 2:**
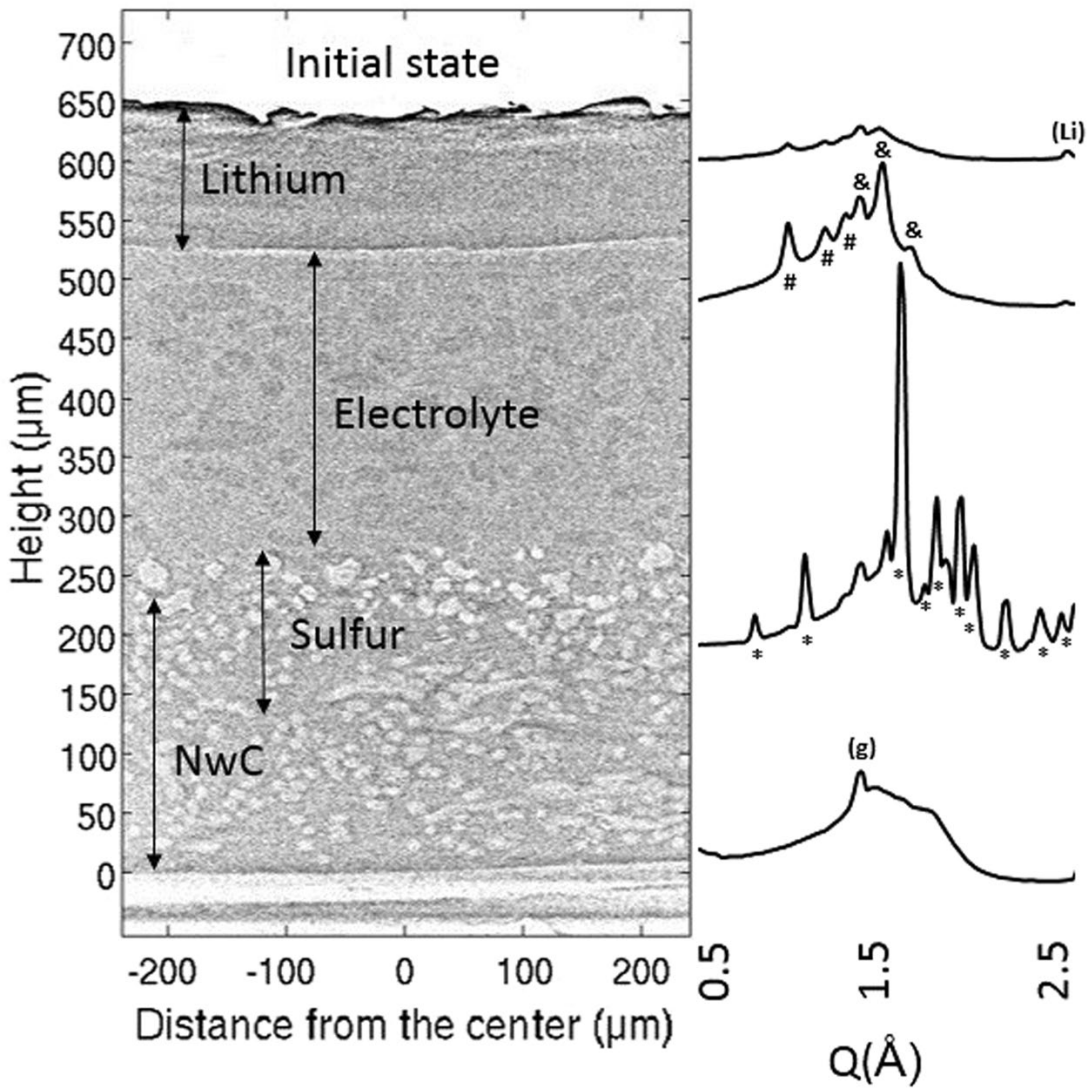
Vertical slice of the tomographic cell at initial state with the associated X-Ray Diffraction (XRD) patterns ((g): graphite//*: sulfur//#: celgard ®//&: viledon ®//(Li): lithium). Light grey corresponds to high absorptive species (*i*.*e*. NwC & sulfur), whereas dark grey corresponds to less absorptive species (*i*.*e*. electrolyte & lithium).

Tomography gives information about the X-ray attenuation coefficient of the sample, but is insensitive to crystallinity and to the particular combination of chemical species and macroscopic density leading to a given absorption. Details of sub-micron particles are not visible due to the resolution of the system used for the tomography measurement. XRD, on the other hand, is sensitive to the different crystalline phases present in the sample. Coupling the two techniques therefore can give a complete picture of a sample over a large length scale, from atomic to macroscopic. Figure 2 shows the interest of combining these methods to characterize the state of the electrochemical cell.

## Discussion

A tomographic slice of the cell in the initial state is shown in Fig. 2. Less absorbing regions are represented by darker color whereas brighter regions correspond to high absorption. The lithium layer can easily be distinguished. In the initial state, this layer is homogeneous in density and relatively flat. The lithium thickness measures 126 µm on average, which is in good agreement with the supplier specifications (135 µm). Sulfur is initially deposited on top of the NwC, due to the coating process, with particle diameters on the order of tens of micrometers. Thus the relevant features in the cell can be distinguished in spite of the low absorption of the constituent species and the high energy of the incident radiation.

XRD patterns taken all along the height of the cell in the initial state are shown in Fig. 2. At the bottom of the cell (20 µm height), a peak due to graphitic carbon at 1.39 Å (marked by (g)) is detectable only, and attributed to the NwC current collector. Higher in the cell (220 µm), the characteristic pattern of orthorhombic α-sulfur (labeled with *) can be seen. Above, the sulfur peaks vanish while the peaks from the components of the electrolyte layer (500 µm height), both Viledon® (#) and Celgard® (&) appear and persist until one observes the lithium layer marked by the peak (Li) at 2.54 Å.

**Figure 3:**
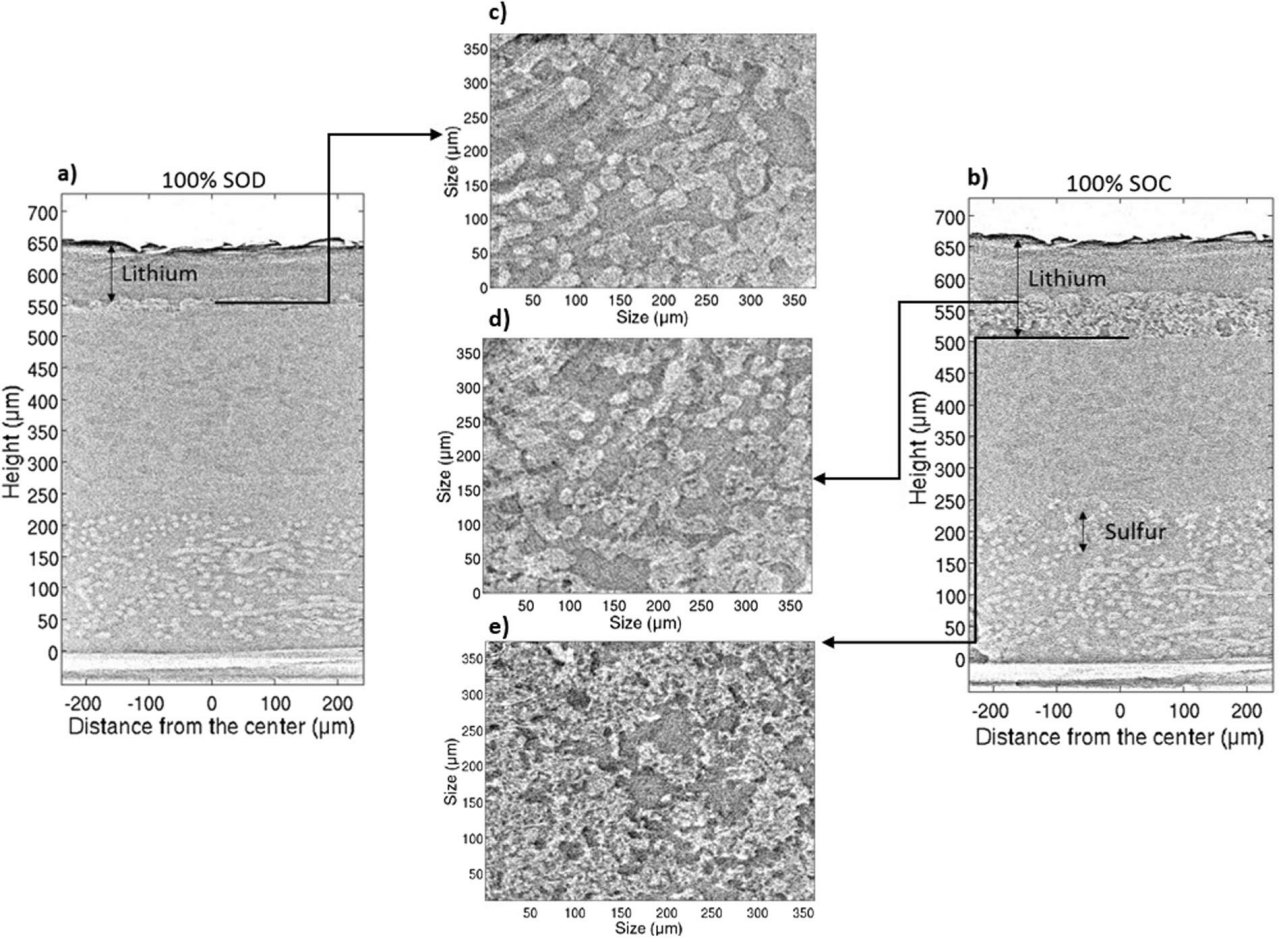
(**a**) Vertical slice of the cell at the end of the first discharge (100% SOD). (**b**) Vertical slice of the cell at the end of the first charge (100% SOC). (**c**) Horizontal slice of the cell at 100% SOD at 560 µm height. (**d**) Horizontal slice of the cell at 100% SOC at 570 µm height. (**e**) Horizontal slice of the cell at 100% SOC at 505 µm height.

By comparing the 3-D tomographic reconstruction and XRD patterns in the initial state, it is thus possible to associate all of observed morphological objects with the corresponding chemical species. The cell was then cycled, and X-ray diffraction patterns and absorption data were measured *operando* as described above. Two vertical slices of the 3-D tomographic reconstructions at different states of charge are presented in Fig. 3a,b.

The most obvious difference in the morphology of the cell components between the first discharge and the first charge is in the size and homogeneity of the lithium electrode (Fig. 3a,b). At the end of the first discharge, *i*.*e*. 100% state of discharge (100% SOD), due to lithium stripping, the interface between the lithium and the electrolyte is heterogeneous and not flat. However, the lithium layer is homogeneous in term of density. Figure 3c shows that the oxidation of the lithium is localized and not uniform, which creates craters (light grey) and lithium pads (dark grey) corresponding to the non-oxidized lithium. As a consequence, the interface becomes heterogeneous and, as already demonstrated^34^, such interface likely favors nucleation and growth of dendrites during the subsequent recharges. Compared to the initial state (Fig. 2a), the average thickness of lithium has diminished from 126 to 106 µm (20 ± 3 µm have been consumed), while a stripping of 14 µm thick lithium layer should be expected, according to the coulometry (2.7 mAh.cm^−2^) and considering a uniform oxidation process. This slight difference is consistent with a non-uniform oxidation of lithium layer. At the end of charge (100% SOC, Fig. 3b), the electrodeposited lithium forms a porous region, and a moss of lithium is observed at the interface between the dense native lithium and the electrolyte. Interestingly, the dense lithium is always 106 µm thick, which means that all electrodeposited lithium is found almost entirely in the porous layer (Fig. 3d). At 100% SOC, the lithium layer measures approximately 164 µm, giving an average density of the deposited layer during first charge of just 17%. This value is very low and can easily explain the poor cycling behavior of the lithium anode in such a liquid-based electrolyte. The average density of the redeposited layer during the second cycle is approximately 21%, similar to the first cycle and indicating that the initial lithium morphology is never recovered upon cycling. These morphological changes in the lithium electrode have important consequences for the functioning of Li/S batteries. As deposited lithium is very porous, a strong impact on the reliability and safety^35^ may be expected. Furthermore, the faradic efficiency of a mossy lithium electrode is very poor due to the formation of fresh SEI, which consumes electrolyte on the surface of the moss. As a consequence, rapid lithium electrode fading and poor cyclability may be expected as well, due to the presence of “dead” mossy lithium^36^.

**Figure 4:**
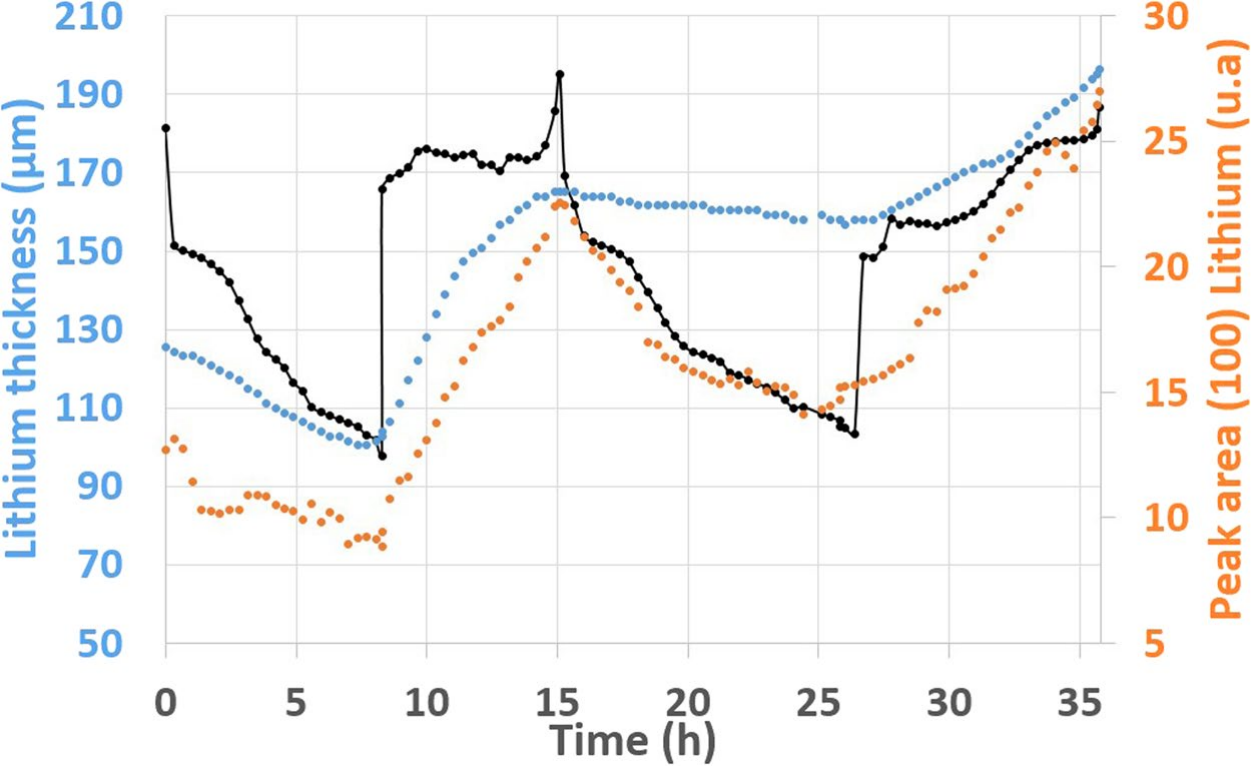
Comparison between tomography and XRD lithium analysis with the associated voltage profile. Lithium thickness was calculated from the tomographic images by counting the number of pixels. Lithium peak area was integrated from XRD pattern at each time.

In order to correlate both tomographic and diffraction data recorded on the lithium layer, a comparison of the ‘apparent’ lithium amount as measured by the two techniques in the electrode was carried out, with the lithium volume determined by imaging compared to the quantity of lithium as measured by the (100) peak area in XRD (Fig. 4).

As expected, the lithium thickness and total quantity as determined by XRD evolve qualitatively in accordance with the voltage profile during the first cycle. For the 2^nd^ cycle, according to tomography data, the lithium thickness diminishes by 8 ± 3 µm during the 2^nd^ discharge, in agreement with what would be expected from the coulometry (i.e. 8.8 µm). This confirms that solely the dense lithium was oxidized electrochemically while the mossy lithium does not participate to the electrochemical reactions. On the other hand, the quantity of lithium in the electrode determined by XRD is significantly reduced. As a result, both the dense and mossy lithium may be oxidized during the discharge. Indeed, without participating to electrochemical reactions, the mossy lithium, with a well-developed surface, reacts strongly with the electrolyte and forms fresh SEI. This chemical oxidation consumes lithium without involving electron in the external electric circuit. Additional experiments are currently ongoing to aim at improving the quantitative analyses of the diffraction data.

In order to perform an analysis of the density over the entire cell and characterize its global evolution while cycling, density averages were taken over regions of 2400 µm^2^ in the x-y plane (parallel to cylinder radius) – Details of the procedure are given in the supplementary information.

**Figure 5:**
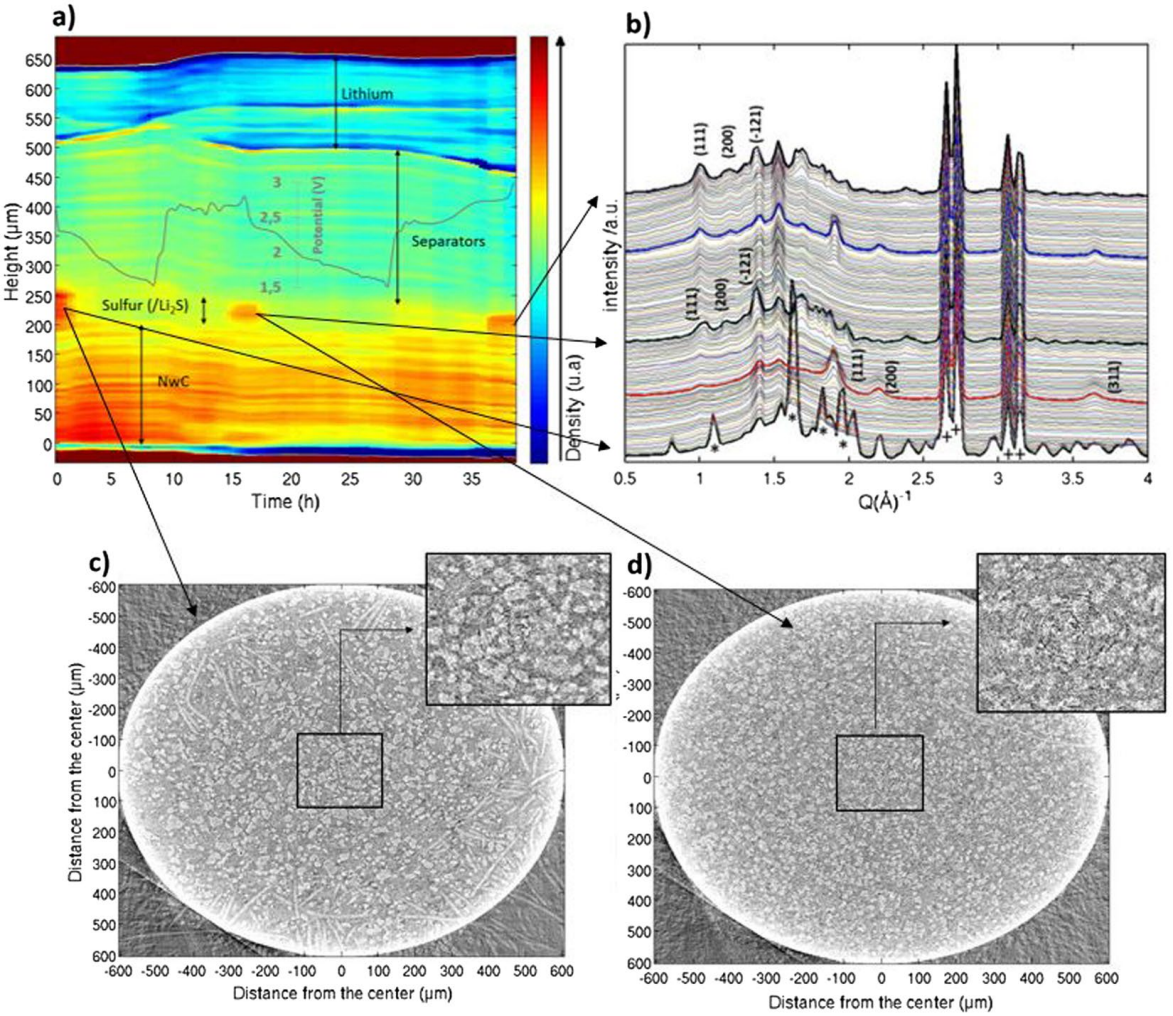
(**a**) Integration over a plane of the 3D tomographic reconstruction showing median pixel values within each vertical layer during the two first cycles. (**b**) Corresponding time evolution of the XRD pattern in the 200 µm slice, corresponding to the top of the NwC. (**c**) Horizontal slice in the carbon binder – sulfur domain, in the initial state and zoom of the 400 µm^2^ indicated. (**d**) Horizontal slice in the carbon binder – sulfur domain, at the end of first charge and zoom of the 400 µm^2^ square indicated.

This can be done without major loss of information, as the battery is to first approximation a 1D object with cylindrical symmetry and the selected region was assumed to be representative of the whole system. A median filter was applied with a radius of 5 pixels so as to reduce the noise in the reconstruction. By plotting the density of the layers over time, the composition of battery components and their positions within the cell could be followed during the two cycles (Fig. 5a–d).

This representation allows us to follow the lithium consumption and deposition processes during cycling and illustrates the negative electrode interface heterogeneity, as previously discussed. In Fig. 5a, the lithium layer clearly displays a strong variation of density at the interface between subsequent layer depositions. This effect is probably accentuated by the fact that no pressure was applied to the cell and thus that the lithium interface was relatively free to evolve without constraints. As already mentioned before, these morphological changes in the lithium electrode have important consequence for the cyclability and reliability of Li/S batteries.

Between the two electrodes, the electrolyte can be divided into two regions: one with a 24 µm thick Celgard® separator, found between the two yellow parts (501 to 525 µm in the initial state), and one with the additional Viledon® separator which measures 220 µm.

The electrolyte is homogeneous while cycling, even if there is a slightly decrease in density which could be interpreted by the electrolyte depletion, probably due to wetting of the lithium layer’s porosity.

In positive electrode, the sulfur is associated with zones of higher density. Semi-quantitative analysis allows the determination of the penetration depth in the non-woven carbon. In the initial state, the sulfur is concentrated on the top of the NwC and penetrates to a depth of approximately 120 µm, a result of the initial coating process. At the end of the first discharge, Li_2_S is distributed over a depth of approximately 180 µm through the NwC. Sulfur grows at the end of the charge^19^, and is also distributed over the same 180 µm depth. However, as the specific surface of carbon material is mainly brought by the initial coating on the top of the electrode, sulfur particles remains always more concentrated on the top of the NwC.

A study of the changes in the XRD pattern of the sulfur electrode upon cycling (Fig. 5b) and an analysis of the phase fractions of sulfur species (shown in the supplementary information), allow a more detailed analysis of the evolution of this region. In the initial state, the diffraction pattern at the electrode contains only orthorhombic α-sulfur peaks (four main peaks: “*”) and the peaks from the aluminum casing (+). During discharge, these peaks decrease linearly with time until their total disappearance at 205 mAh.g_s_^−1^, indicating complete reduction of sulfur, in good agreement with the theoretical capacity of this first region and with the literature data^22^. At this point, all sulfur species exist as soluble lithium polysulfides only^22^. Subsequently, the appearance of Li_2_S is observed, and the quantity of Li_2_S reaches a maximum at the end of discharge (red pattern) before vanishing during charging. Ultimately, only 300 mAh.g_s_^−1^ is obtained during the second discharge plateau, which represents a quarter of the expected capacity on this plateau. This shows that the electrode discharge capacity is limited by the uncomplete deposition of insulating Li_2_S product. Looking at the XRD patterns collected along the cell, it is clear that Li_2_S is also concentrated on the top of the NwC, while no crystalline Li_2_S deposition on the top of to the lithium electrode can be detected in the present experiment. The growth of the Li_2_S is reproducible during the second cycle. When sulfur reappears upon subsequent charging, and in all further cycles, it crystallizes as monoclinic β-S_8._ Moreover, tomographic slices (Fig. 5c and d) show that sulfur particles grow back with a different global morphology. The electrochemical nucleation and growth of the β-S_8_ leads to smaller particles with a narrower size distribution compared to the initial sulfur particles^10^, and presumably the increased surface to volume ratio is the driving force for stabilizing the nominally metastable β-S_8_.

To conclude, we have presented a complete *operando* characterization study of a Li/S cell, by combining electrochemistry with X-ray diffraction and absorption tomography. This unique combination of techniques enables the correlation of the structural and morphological state of the components with electrochemical behavior, allowing the identification of key phenomena in the battery. With X-ray diffraction, the temporal and spatial distribution of lithium, sulfur and Li_2_S can be followed within the cell, while with tomography, the evolving morphology of the cell components is observed. An important observation explaining the functional behavior of these cells concerns “breathing” of the lithium plating/striping. The highly heterogeneous behavior of the lithium plating explains the poorly reversible consumption and deposition on the negative electrode while cycling, and is key to understand the whole system cyclability in these technologies. In this experiment, redeposited lithium was porous and the interface between lithium and electrolyte was found to be quite heterogeneous, which may be related to the lack of pressure applied to the cell. In any case, these results point out the poor lithium metal reversibility in liquid-based electrolyte, and the need to address this issue. In this context, the characterization tools which have been described here seem to be highly relevant. Additional experiments are currently ongoing with an optimized cell design (pressure controlled) that will provide spatial resolution of the chemical composition in the entire cell.

On the positive electrode, the quantity of sulfur decreases linearly with time until 25% SOD, then regrows in the β-form. This β-sulfur grows in smaller particles more sparsely distributed in the NwC with respect to the initial sulfur, as well as preferentially being deposited on the surface of the NwC electrode. As a continuation of this work, sulfur active material and counterparts will be monitored into the electrode depth during cycling, which can allow for designing improved cathode structures.

Finally, the combination of electrochemistry, absorption tomography and XRD gives a clear and detailed view of electrochemical cell components, allowing the correlation of macroscopic and microscopic phenomena in whole batteries under true *operando* conditions. By combining these tools, we can map the complex state of active materials in the entire cell, and correlate the physical state with electrochemical behavior. In particular, this allows us to demonstrate notably that the negative lithium electrode remains one of the main challenges of such technology. We believe that this coupled approach will permit to get a deeper understanding of the performance limiting degradation phenomena that occur in batteries in general.

## Methods

Positive electrodes were made of carbon-based current collector (porous non-woven carbon paper, H2315 ®, Freudenberg), with a high sulfur loading (≈3.9 mgsulfur.cm^−2^). Super P® (Timcal) and PVdF 5130 (Solvay; 12 wt% solution in N-methyl-2-pyrrolidinone, NMP) were used as a conductive carbon and a binder, in the weight ratio of 80/10/10 wt% (S_8_/SuperP/PVdF). A mixture of this species was prepared with a small amount of cyclohexane. After homogenization, the mixture was coated, using a doctor blade, on the current collector before being dried at 55 °C during 24 h in an oven.

The cell was prepared in an aluminum crucible with a diameter of 6.7 mm and a height of 5 mm. An insulating layer of Kapton® was taped inside the aluminum container. The cell was assembled in a dry room (−40 °C dew point) using a positive electrode (described above, soaked with organic electrolyte), a lithium foil (135 µm, Rockwood Li) and a porous separator (Celgard® 2400) with Viledon® also soaked with organic electrolyte (Fig. 1a). Electrolyte was composed of 1 mol.L^−1^ of LiTFSi (Aldrich) + 0.1 M LiNO_3_ in a mixture of tetraethylene glycol dimethyl ether (TEGDME; Aldrich) and 1,3-dioxolane (DIOX; Aldrich) with a 50/50 volume ratio.

Electrochemical tests were carried out with VMP® biologic in a voltage range 1.5–3.0 V at the current rates of C/20 (≈0.33 mA.cm^−2^) and C/40 (≈0.17 mA.cm^−2^). The first cycle was performed at C/20 and the second at C/40.

*In situ* and *operando* XRD and X-ray absorption measurements were carried out at beamline ID15A at the European Synchrotron Radiation Facility (ESRF: Grenoble, France) with a monochromated incident energy of λ = 0.1778 Å (69.7 keV). XRD patterns and absorption tomography data were recorded alternatively with a recording frequency of ≈20 min. The time for switching geometry and measure the absorption tomography took 5 min. The beam size was varied around 1.5 * 1.5 mm^2^ for tomography, and 20 * 20 µm^2^ full width at half maximum (FWHM) for diffraction, by inserting X-ray compound refractive lenses into the beam. Due to the large size of the cell, only local tomography was carried out on a cylinder in the center with a diameter 1210 µm, corresponding to the size of the detector used. The entire active height of the cell (~650 µm) was measured. The image resolution for the tomography was 1.2 * 1.2 * 1.2 µm^3^ per voxel, corresponding to the size of a detector pixel. X-ray diffraction beam was carried out over the entire height of the active part of the cell, in 20 µm steps. Lithium thickness was measured by counting the number of pixel in the layer from the integrated 3D tomographic reconstruction.

A prototype CdTe Pilatus detector from Dectris was used for the diffraction experiments, and a Dalstar 1M60 CCD-camera coupled to high resolution phosphor was used for imaging.

## Electronic supplementary material

Supplementary Information
