# Supplementary material for: Multiscale characterization of a lithium/sulfur battery by coupling operando X-ray tomography and spatially-resolved diffraction
Source: Sci Rep. 2017 Jun 5;7:2755. doi: 10.1038/s41598-017-03004-4 (PMC5459854; doi:10.1038/s41598-017-03004-4)
Supplement: Supplementary file 1 — Supplementary Information [file 41598_2017_3004_MOESM1_ESM.pdf]

Supplementary Information

Multiscale characterization of a lithium/sulfur battery by coupling *operando* X-ray tomography and spatially-resolved diffraction.

*Guillaume Tonin, Gavin Vaughan, Renaud Bouchet, Fannie Alloin, Marco Di Michiel, Laura Boutafa, Jean-François Colin, Céline Barchasz**

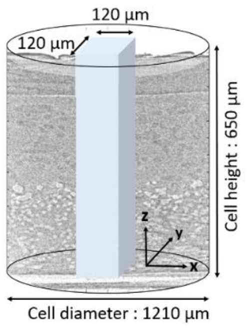

Figure S1. Schematic representation of volume integration. The blue cylinder corresponds to the section taken for the density integration.

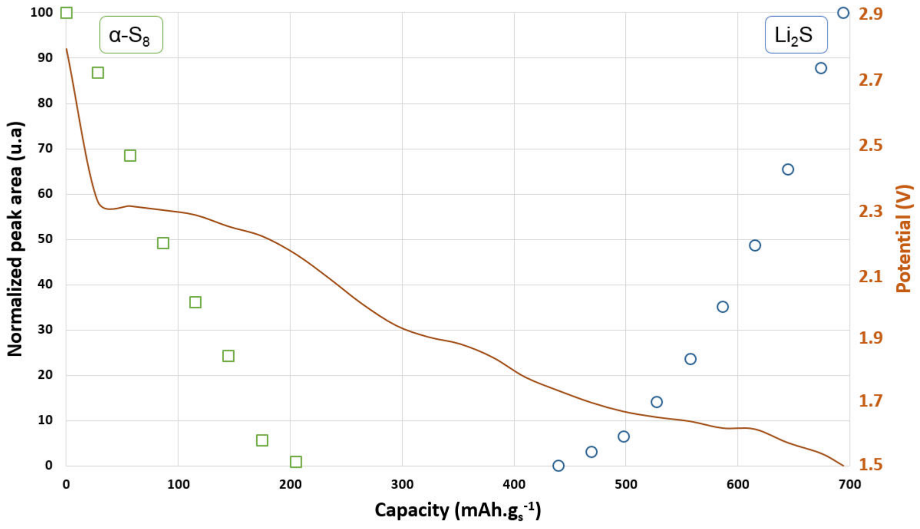

Figure S2. Phase fraction of sulfur species in the sulfur positive electrode as a function of the capacity of the cell.
